# Production and Characterization of Exopolysaccharide From Newly Isolated Marine Probiotic *Lactiplantibacillus plantarum* EI6 With *in vitro* Wound Healing Activity

**DOI:** 10.3389/fmicb.2022.903363

**Published:** 2022-05-13

**Authors:** Eman H. Zaghloul, Mohamed I. A. Ibrahim

**Affiliations:** National Institute of Oceanography and Fisheries, NIOF, Egypt

**Keywords:** exopolysaccharide, Lactiplantibacillus, wound healing, probiotics, chemical characterization

## Abstract

Because of its safety, biological activities, and unique properties, exopolysaccharide (EPS) from lactic acid bacteria (LAB) has been developed as a potential biopolymer. A few studies have investigated the EPS produced by marine LAB. This study reports the wound healing activity of an EPS produced by a marine isolate identified as *Lactiplantibacillus plantarum* EI6, in addition to assessing *L. plantarum* EI6's probiotic properties. EI6 demonstrated promising antimicrobial activity against different pathogenic bacteria, as well as the ability to withstand stomach pH 3, tolerate 0.3% bile salt concentration, and exhibit no signs of hemolysis. Furthermore, EI6 was able to produce 270 mg/L of EPS upon growth for 48 h at 37°C in an MRS medium enriched with 1.0% of sucrose. The chemical features of the novel EI6-EPS were investigated: the UV-vis estimated a high carbohydrate content of ~91.5%, and the FTIR emphasized its polysaccharide nature by the characteristic hydroxyl, amide I, II, & III, and glycosidic linkage regions. The GC-MS and NMR analyses revealed the existence of five monosaccharides, namely, rhamnose, galactose, mannose, glucose, and arabinose, existing mainly in the pyranose form and linked together by α- and β-glycosidic linkages. EI6-EPS was found to be safe (IC50 > 100 μg/ml) and induced human skin fibroblasts (HSF) proliferation and migration. These findings imply that EI6 can be used as a safe source of bioactive polymer in wound care.

## Introduction

The skin is the body's largest organ. It serves as a barrier and is the first line of defense against pathogens entering the body. The skin's microbiota maintains its health and homeostasis. Undesirable alternations of this homeostasis occur daily due to accidental burns, inflammation, wounds, and surgeries. In healthy individuals, wounds heal normally in a few days, but in some cases, such as in diabetic patients, some wounds take longer to heal or do not heal at all (Mohammed et al., 2016). Infection of the wound is a severe complication in such cases, further delaying natural wound healing (Wei et al., 2019). These patients benefit significantly from the use of topical treatments to accelerate wound healing (Yates et al., 2012). Many of the available wound healing drugs are costly and induce several side effects. Therefore, there is a constant demand for effective wound healing bioactive compounds of natural origins that are safe, effective in cost, and can be better tolerated by patients (Demirci et al., 2014; Okur et al., 2020). Curcumin, quercetin, essential oils, lawsone, resveratrol, aloe vera, andrographolide, bilirubin, and astragaloside are some bioactive compounds that have demonstrated a significant wound healing potential (Kant et al., 2021).

Since the ocean covers more than 70% of the earth's surface, a diverse range of marine species provides a plentiful supply of natural products, and the relevance of marine organisms as a source of new bioactive compounds is proliferating (Lindequist, 2016). The sea provides a tremendous resource for new chemicals, with marine organisms accounting for almost half of the world's biodiversity (López-Abarrategui et al., 2012). Furthermore, marine organisms have produced different types of substances for a variety of reasons, including the fact that they live in a very demanding, competitive, and aggressive environment, which is very different in many ways from the terrestrial environment and necessitates the production of very specific and potent active molecules (Demain et al., 2017,Pham et al., 2019).

Exopolysaccharides (EPS) is a group of high molecular weight biopolymers produced during the metabolic process of some microorganisms such as bacteria and fungi (Guo et al., 2013). Since EPS can be composed of one or more types of monosaccharides, they are classified as homopolysaccharides or heteropolysaccharides (Min et al., 2019). They can also be produced, either attached to the microorganism cell surface, forming a capsule, or released into the surrounding environment (Mazzoli et al., 2014). Bacteria are the most commonly used source for EPS production as they replicate rapidly, and the EPS forms loosely attached mucoid layers that can be easily separated from cells by any EPS isolation methods. They are nontoxic, biocompatible, and biodegradable (Angelin and Kavitha, 2020).

Moreover, each bacterial strain produces distinct EPS with different biological activities, and bacterial EPS can be utilized alone or in combination with other materials for a wide range of applications in the biomedical and pharmaceutical fields (Abdelhamid et al., 2020). The vast applications of bacterial EPS can be explained by the large number of derivatives obtained by controlling production parameters. The producing strain, the culture media composition, and culture conditions can affect the quantity, chemical structure, and bioactivity of the produced EPS (Yilmaz et al., 2015). Therefore, careful selection of the EPS-producing strains, optimization of production conditions, and detailed information about the EPS structure are significant for novel EPS production with prominent biological activities (Bachtarzi et al., 2020).

One of the main problems that prevents the large-scale manufacturing and commercialization of certain EPS is the pathogenicity of some of the producing strains (Costa et al., 2018). On the contrary, probiotic bacteria such as *Lactobacillus, Bifidobacterium, Lactococcus*, and *Streptococcus* are generally regarded as safe, so they have been widely used for EPS production. Moreover, they can survive in gastric conditions such as high bile salt concentrations and low pH, in addition to managing to colonize the intestine. Recent studies have suggested that applying lactic acid bacteria (LAB) to the skin enhances skin health and contributes to fighting diseases. Lactobacilli strains have been proven to aid in wound healing, resistance to pathogens, and the recovery of skin inflammations. *Lactobacillus* sp. represents a large group of potential probiotic bacteria, and they are found in a variety of nutrition-rich habitats, including food and feed, and the gastrointestinal tracts of humans, fish, and animals (Duar et al., 2017; Brandi et al., 2020).

The probiotic EPS has been shown to act as a prebiotic that enhances the growth and colonization of favorable bacteria in the human gastrointestinal tract (Welman and Maddox, 2003). Some probiotic species have been proven to enhance wound repair in the gastrointestinal tract in different *in vitro* and *in vivo* studies (Lukic et al., 2017). For instance, in the presence of *L. rhamnosus* GG and *L. gasseri*, the healing of stomach ulcers in rats is thought to be hastened. As probiotics have been shown to aid in the healing of gastric wounds, researchers have begun to investigate if they might aid in the healing of cutaneous wounds as well (Sultana et al., 2013). At the same time, probiotic derivatives may be more suitable for wound healing applications, as there is no need to overcome the obstacles concerning maintaining the viability of living cells.

Therefore, this study aims to isolate EPS-producing marine LAB, assess its probiotic potential, produce a novel safe EPS with wound healing activity, and characterize its structural properties using various techniques.

## Materials and Methods

### Isolation and Identification of EPS-Producing Marine LAB Strain

Marine shrimp samples (5 samples) were used to isolate an EPS-producing LAB. The samples were dissected, and serial dilutions of the fragmented guts were prepared using sterile saline. Subsequently, 1 ml of each dilution was inoculated into De Man, Rogosa, and Sharpe (MRS) medium (Lab M, UK) agar plates and incubated at 37°C for 48 h under anaerobic conditions (candle jar) (Amiza et al., 2006). Mucoid colonies were picked up for purification by successive streaking on MRS agar plates (Abid et al., 2018). Gram-positive, catalase-negative, non-motile, and non-spore-forming isolates were stored in MRS broth with glycerol at 20% (v/v) at −20°C for further studies.

Of the obtained isolates, four, namely, EI6, EI7, EI8, and EI9, were grown in MRS broth (200 ml) supplemented with 1.0% sucrose sugar for 48 h at 37°C. The cultures were then filtered through a bacterial filter to remove bacterial cells. For protein degradation, trichloroacetic acid (TCA, 10% w/v) was added to the supernatant for 30 min. Then, the culture supernatants were centrifuged at 5,000 rpm for 15 min, and the pellets were discarded. Next, cold absolute ethanol was added to the culture supernatants (3:1 v/v) and stored for 48 h at 4°C. The supernatants were centrifuged at 5,000 rpm for 15 min, and the precipitates were recovered and dialyzed against distilled water overnight through bags with a pore size of 12 kDa (Sigma, USA). Afterward, the obtained EPS was dried overnight at 30°C, and the dry weight was determined as the mean ± SD of three independent experiments (Amer et al., 2020).

The isolate EI6 with the highest EPS yield was investigated under a scanning electron microscope (SEM) and characterized biochemically by the VITEK 2 system version 07.01 (BioMerieux, France) at Mabaret El Asafra Laboratories, Egypt. Moreover, the bacterial isolate was identified by 16s rRNA gene sequencing analysis. Total DNA was isolated by the DNA isolation kit (Qiagen, Germany) as described by the manufacturer. The isolated DNA was visualized by ethidium bromide staining after electrophoresis in a 1.0% agarose gel, and it was amplified by PCR using the primer pairs (Uni27F, 5′-AGAGTTTGATCCTGGCTCAG-3′ and Uni1492R, 5′-GGTTACCTTGTTACGACTT-3′) under the following conditions: 3 min of denaturation at 94°C, followed by 35 cycles of amplification at 94°C for 30 s, 55°C for 30 s, and 72°C for 1 min. Final extension was allowed at 72°C for 10 min. The PCR product was sequenced by the Applied Biotechnology Company, Egypt. The obtained sequence was analyzed using BLAST, and the phylogenetic analysis was performed using the facilities provided by the website http://www.phylogeny.fr/.

### Assessment of the Probiotic Potential of EI6

#### Antimicrobial Activity

The antimicrobial activity of EI6 was evaluated using an agar well-cut diffusion test against the indicator pathogens (*Pseudomonas fluorescens* ATCC 13525, *Streptococcus agalactiae* ATCC 13813, *Aeromonas hydrophila* ATCC 13037, *Staphylococcus aureus* ATCC 25923, *Escherichia coli* ATCC 8739, *Enterococcus faecalis* ATCC 29212, and *Klebsiella pneumonia* ATCC 13883). In brief, 50 μl of 10^6^ CFU/ml test pathogen was inoculated into nutrient agar plates, wells of 8 mm in diameter were cut into the agar, and 100 μl of cell-free culture supernatant of EI6 (adjusted to pH 6.5 with 1 M NaOH) was added and stored at 4°C for 1 h to allow diffusion, and then the plates were incubated at 37°C for 24 h. Then, the diameter of the inhibition zones around the wells was measured (Zaghloul and Ibrahim, 2019).

#### Blood Hemolysis

Blood hemolytic activity of isolate EI6 was evaluated by streaking on blood agar plates (5.0% sheep blood) and observation of any signs of hemolysis (darkening: α-hemolysis, clear zone: β-hemolysis, and no change: γ-hemolysis) after 24 h of incubation at 37°C (Guttmann and Ellar, 2000).

#### Antibiotic Susceptibility

Antibiotic susceptibility was evaluated as described by Abid et al. (2018). Briefly, overnight culture of EI6 (10^6^ CFU/mL) was inoculated on MRS agar plates. Antibiotic disks (Oxoid, UK) were added to the agar surface and incubated for 24 h at 37°C. Susceptibility was detected by the presence of an inhibition zone around the disk.

#### Low pH Resistance

The isolate EI6 was tested for its potential to withstand low pH values following the method described by Balamurugan et al. (2014). Briefly, different MRS broths adjusted to pH 6.4, 4.0, 3.0, and 2.0 were inoculated with 1 ml of 10^6^ CFU/ml of an overnight culture of EI6 and incubated for 24 h at 37°C. Bacterial growth was determined by measuring the optical density at 620 nm using a spectrophotometer (Unico, USA) at 1, 2, 3, 4, 5, 6, and 24 h.

#### Bile Salts Resistance

The ability of isolate EI6 to resist high bile salt concentrations was evaluated. For this purpose, an aliquot of 25 ml of MRS broth supplemented with varying concentrations of bile salts (0, 0.1, and 0.3%) was inoculated with 1.0 ml of 10^6^ CFU/ml overnight culture of EI6 and incubated for 24 h at 37°C. Bacterial growth was monitored by measuring the optical density at 620 nm using a spectrophotometer (Unico, USA) at 1, 2, 3, 4, 5, 6, and 24 h (Patel et al., 2014).

#### Mass Production of EPS

The marine bacterial isolate EI6 was used for the mass production of EPS. Isolate EI6 was cultured in 2,000 ml of MRS broth medium supplemented with 1.0% sucrose sugar (w/v) and incubated at 37°C for 48 h. Subsequently, the EPS was recovered, as previously stated.

### Physicochemical Characterization of EI6-EPS

#### Total Sugar Content

The total carbohydrate content of the purified EI6-EPS was determined using the recommended method by Dubois et al. (1956). A 500 μl of EI6-EPS solution (5 mg/ml) was treated successively with 500 μl of phenol (2.5%; w/v), and 2.5 ml of sulfuric acid (>99.0%). The reaction mixture was then incubated at ambient temperature for 15 min before measuring the absorbance at 490 nm using a UV-vis spectrophotometer (JANEWAY 6800, UK). The carbohydrate content was expressed as glucose%, compared with the glucose standard curve (10–100 μg/ml) (Sran et al., 2019).

#### Structural Functionalities

The purified EI6-EPS powder was subjected to FTIR spectroscopy analysis to identify the major and characteristic functional groups. The purified EI6-EPS powder was practically loaded over the single crystal germanium of the FTIR spectrometer (Bruker, ALPHA, Germany) equipped with the attenuated total reflectance (ATR) technique. After background subtraction, the spectrum was recorded between 4,000 and 400 cm^−1^ with a resolution of 4.0 cm^−1^ and 64 scans (Amer et al., 2020; Ji et al., 2021, 2022).

#### Structural Studies

^1^H NMR experiment was operated on a JEOL-Ltd spectrometer (Japan, 500 MHz) for the structural studies of the EI6-EPS. Approximately 20 mg of the lyophilized powder was completely dissolved in 0.75 ml DMSO-*d*_6_ under gentle warming. The ^1^H NMR spectrum was recorded at 323 K, and the chemical shifts were expressed in ppm (δ). The data were analyzed using the MestReNova version 6.0.2-5475 (©2009 Mestrelab Research S.L.) software (Trabelsi et al., 2017).

#### Monosaccharide Composition

The sugar moieties of the purified EI6-EPS were identified as silylated glycosides through the following three consecutive steps: acid hydrolysis, silylation, and then identification using gas chromatography-mass spectrometry (GC-MS) (Chaplin, 1982; Dueñas-Chasco et al., 1997; Mao et al., 2006; Ruiz-Matute et al., 2011). About 20 mg of the EI6-EPS powder was hydrolyzed with 3 ml of sulfuric acid (2 M), heated at 105°C for 10 h, and then the tube was cooled at ambient temperature before neutralization with barium carbonate. The formed precipitates were removed by centrifugation, while the supernatant was filtered through a 20-μm syringe before lyophilization. The dried hydrolysates were silylated by adding 1:1 pyridine-BSTFA (*N,O*-bis(trimethylsilyl) trifluoroacetamide) for 16 h at 80°C (50 μl/mg dried sample) (Chaplin, 1982; Ruiz-Matute et al., 2011). A 2 μl of the derivatized sugars were injected into GC-MS (MassHunter GC-MS 1989-2014, Agilent Technologies, Inc.) following a previous separation method. The detector and injector temperatures were maintained at 320°C, and the column HP5MS (30 m × 0.25 mm × 0.25 μm) was first set at a temperature 100°C for 1 min and then ramped from 100 to 260°C at 4°C for 1 min, then held at 260°C for 10 min. The carrier helium gas was set at a flow rate of 1 ml/min (Ben Gara et al., 2017). Finally, the identification of the monosaccharides was based on the NIST library (Olasehinde et al., 2019).

#### Morphological and Elemental Studies

The SEM images of the EI6-EPS were captured by a scanning electron microscopy spectrometer (SEM, JSM-IT 200, Jeol, Japan). First, the sample was coated with gold (15 Å) for 2 min by physical vapor deposition before visualization at an accelerating voltage of 20.0 kV (Ibrahim et al., 2020). The elemental composition analysis of EI6-EPS was then performed without any pretreatment using a scanning electron microscope-energy dispersive X-ray (SEM-EDX) spectrometer. The emitted X-rays were utilized to calculate the weight and atomic percentages of the recorded elements (Kavita et al., 2014).

#### Cytotoxicity Assay

Human dermal fibroblast (HDF) was obtained from Nawah Scientific Inc. (Mokatam, Cairo, Egypt). Cells were maintained in Dulbecco's Modified Eagle Medium (DMEM; purchased from Sigma-Aldrich, USA) supplemented with 100 mg/ml of streptomycin, 100 units/ml of penicillin, and 10% of heat-inactivated fetal bovine serum in a humidified, 5.0% (v/v) CO_2_ atmosphere at 37°C.

The sulforhodamine B (SRB) assay was used to determine cell viability. Aliquots of 100 μl cell suspension (5 × 10^3^ cells) were added to 96-well plates and incubated in complete media for 24 h. Cells were treated with another aliquot of 100 μl media containing varying concentrations of EI6-EPS. After 72 h of EI6-EPS exposure, cells were fixed by replacing media with 150 μl of 10% TCA and incubated at 4°C for 1 h. The TCA solution was removed, and the cells were washed five times with distilled water. Aliquots of 70 μl SRB solution (0.4% w/v) were added and incubated in a dark place at room temperature for 10 min. Plates were washed three times with 1.0% acetic acid and allowed to air-dry overnight. Then, 150 μl of TRIS (10 mM) was added to dissolve protein-bound SRB stain; the absorbance was measured at 540 nm using a BMG LABTECH®-FLUOstar Omega microplate reader (Ortenberg, Germany) (Skehan et al., 1990; Allam et al., 2018).

#### *In vitro* Wound Healing Activity

For the scratch wound assay, HSF cells were plated at a density of 3 × 10^5^ CFU/well onto a coated 6-well plate and cultured overnight in 5.0% FBS-DMEM at 37°C and 5.0% CO_2_. The following day, horizontal scratches were introduced into the confluent monolayer. The plate was washed thoroughly with PBS, control wells were replenished with a fresh medium, and the second set of wells was treated with fresh media containing EI6-EPS. Images were taken using an inverted microscope at 0, 24, and 48 h time intervals. The plate was incubated at 37°C with 5.0% CO_2_ in-between time points. The acquired images were analyzed by the MII ImageView software version 3.7. The wound closure % was calculated according to the formula:


$$\begin{array}{l} \text{Wound closure }\%=\;\frac{A_{0}-\;A_{t}}{A_{0}}\;\times \;100 \end{array}$$

where *A*_0_ and *A*_*t*_ are the average areas of the wound measured immediately after scratching (time = zero) and after time = *t* in hours, respectively (Li et al., 2019).

### Statistical Analysis

All experiments were conducted in triplicate, and the results were represented as mean ± standard deviation (SD). Statistical analysis was performed using Microsoft Excel 2010, and the statistical difference was assessed using one-way analysis of variance (ANOVA). Differences at *P* < 0.05 were considered statistically significant.

## Results and Discussion

### Isolation, Biochemical Characterization, and Identification of EPS-Producing Marine LAB

A total of four EPS-producing LAB strains (EI6, EI7, EI8, and EI9) were isolated from the guts of marine shrimp samples as they showed mucoid colonies on MRS media (Guérin et al., 2020). These four isolates were examined for EPS production yield by growing in MRS media supplemented with 1.0% sucrose; they gave EPS yields of ~230, 171, 152, and 205 mg/L, respectively. Accordingly, isolate EI6 was selected for further studies as it gave the highest EPS yield of about 230 ± 1.55 mg/L.

**Figure 1:**
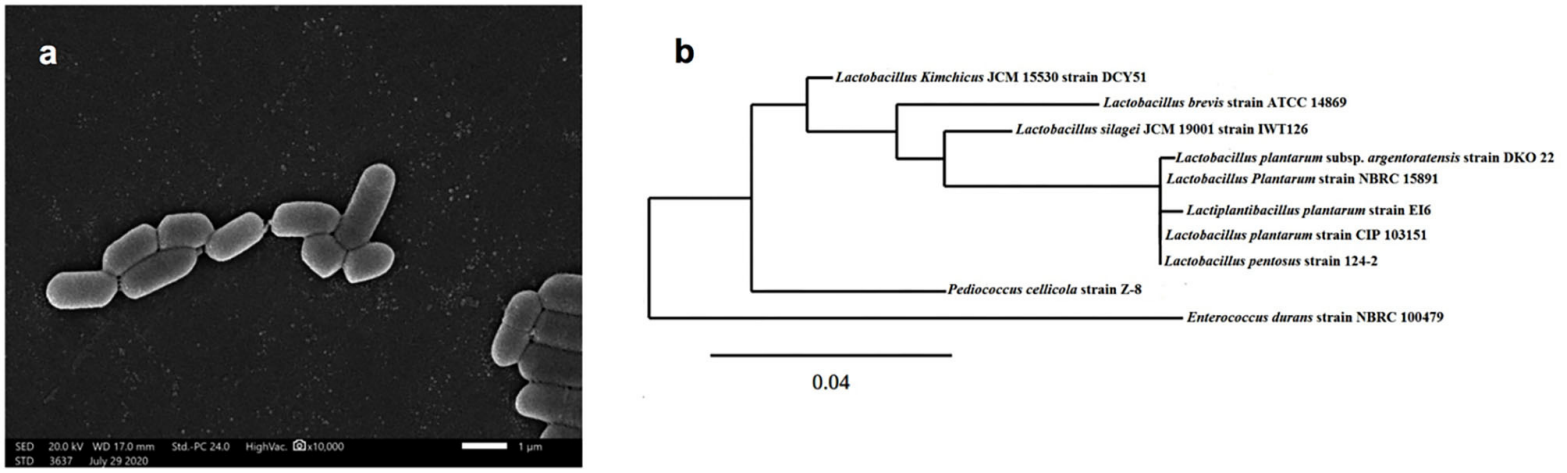
Scanning electron microscope of isolate EI6 **(A)**, and phylogenetic analysis of the marine isolate *Lactiplantibacillus plantarum* EI6 based on 16s rRNA gene sequence **(B)**.

Isolate EI6 (Figure 1A) appeared as gram-positive, non-spore-forming rods, and the examination under the SEM revealed its characteristic rod cell shape. Moreover, it was further characterized biochemically using the VITEK 2 system (BioMérieux, France), as described in Table 1. The VITEK 2 characterization of the isolate EI6 implied certain biochemical properties, including resistance to four antibiotics, namely, Bacitracin, Novobiocin, Polymixin, and Optochin, and the ability to grow in 6.5% NaCl. The growth in salty media is a desired feature for starter cultures, as NaCl is one of the most significant additions for food preservation (Abid et al., 2018).

**Table 1:** Isolate EI6 biochemical characterization using VITEK® 2 system version 07.01 (BioMerieux, France).

**Test**	**Result**	**Test**	**Result**
D-amygdalin (AMY)	+	D-galactose (dGAL)	+
Phosphatidylinositol phospholipase c (PIPLC)	–	D-ribose (dRIB)	+
D-xylose (dXYL)	–	L-lactate alkalinization (ILATK)	–
Arginine dihydrolase 1 (ADH1)	–	Lactose (LAC)	+
Beta-galactosidase (BGAL)	–	N-acetyl-d-glucosamine (NAG)	+
Alpha-glucosidase (AGLU)	+	D-maltose (dMAL)	+
Ala-phe-pro arylamidase (APPA)	–	Bacitracin resistance (BACI)	+
Cyclodextrin (CDEX)	–	Novobiocin resistance (NOVO)	+
L-aspartate arylamidase (AspA)	–	Growth in 6.5% NaCl (NC6.5)	+
Beta galactopyranosidase (BGAR)	–	D-mannitol (dMAN)	+
Alpha-mannosidase (AMAN)	–	D-mannosE (dMNE)	+
Phosphatase (PHOS)	–	Methyl-b-d-glucopyranoside (MBdG)	+
Leucine arylamidase (LeuA)	+	Pullulan (PUL)	–
L-proline arylamidase (ProA)	–	D-raffinose (dRAF)	+
Beta glucuronidase (BGURr)	–	O/129 resistance (comp.vibrio.) (O129R)	+
Alpha-galactosidase (AGAL)	–	Salicin (SAL)	+
L-pyrrolydonyl-arylamidase (PyrA)	–	Saccharose/Sucrose (SAC)	+
Beta-glucuronidase (BGUR)	–	D-trehalose (dTRE)	+
Alanine arylamidase (AlaA)	+	Arginine dihydrolase 2 (ADH2s)	–
Tyrosine arylamidase (TyrA)	+	Optochin resistance (OPTO)	+
D-sorbitol (dSOR)	+	Urease (URE)	–
Polymixin b resistance (POLYB)	+		

The molecular identification of isolate EI6 was carried out through 16s rRNA gene sequencing. EI6 was identified as *Lactiplantibacillus plantarum* with an identity percentage of 99%. The obtained sequence was submitted to the GenBank under the accession number MW413308, and the phylogenetic relationship of the marine isolate EI6 and its close relatives in the NCBI database is demonstrated in Figure 1B.

### Assessment of the Probiotic Potential of EI6

#### Blood Hemolysis

The growth of isolate *L. plantarum* EI6 was examined on blood agar, and it gave no signs of hemolysis, which implies the safety of isolate EI6 to be used as a potential probiotic and its suitability for biotechnological and industrial applications (Nakajima et al., 2003).

#### Antimicrobial Activity

Lactic acid bacteria produce several antimicrobial agents that give it considerable advantages in competition with the other harmful and pathogenic bacteria in the gut. For probiotic bacteria to be able to colonize the gastrointestinal tract, it is essential to have the capability to eliminate the competitors. Therefore, the antimicrobial activity of the marine *L. plantarum* EI6 supernatant was evaluated using an agar well-cut diffusion test against seven indicator pathogens, and the results are represented in Table 2. The data showed that *L. plantarum* EI6 has potent antimicrobial activity with different inhibitory potential, as *Pseudomonas fluorescens* was the most affected pathogen with an inhibition zone diameter of 2.4 cm, and the least activity was detected against *Staphylococcus aureus* with an inhibition zone diameter of 1.2 cm, while no any activity was detected against *Klebsiella pneumonia* or *Escherichia coli*. This activity can be attributed to the antimicrobial substances produced by these LAB strains. These antimicrobial agents include organic acids (e.g., lactic acid and acetic acid), fatty acids, hydrogen peroxide, acetoin, diacetyl, and, most importantly, inhibitory peptides known as bacteriocins (Leblanc and Todorov, 2011). Similarly, the broad spectrum of antimicrobial activity of *Lactiplantibacillus* strains has been reported (Lavilla-Lerma et al., 2013).

**Table 2:** Antimicrobial activity of *L. plantarum* EI6 cell-free supernatant against indicator pathogens.

**Pathogen**	**Inhibition zone** **diameter (cm)**
*Pseudomonas fluorescens* ATCC 13525	2.4 ± 0.05
*Streptococcus agalactiae* ATCC 13813	1.9 ± 0.2
*Aeromonas hydrophila* ATCC 13037	1.5 ± 0.09
*Staphylococcus aureus* ATCC 25923	1.2 ± 0.13
*Escherichia coli* ATCC 8739	0.0
*Enterococcus faecalis* ATCC 29212	1.9 ± 0.23
*Klebsiella pneumonia* ATCC 13883	0.0

#### Antibiotic Susceptibility

Antibiotic susceptibility is a significant criterion for probiotic selection, since their ability to resist some antibiotics may be beneficial (Onyibe et al., 2013). Antibiotic resistance may help them survive in the gut, especially when employed to reestablish intestinal bacteria balance and a healthy environment following antibiotic therapy. LAB in the gut or food products can also serve as a reservoir for antibiotic resistance genes because they may carry plasmids and transposons that encode antibiotic resistance genes. These genes could be transferred to other harmful microbes in the gut or the food chain (Huys et al., 2013). Therefore, before the use of a strain of LAB as a potential probiotic, antibiotic resistance screening must be done to ensure its safety for application.

The disc diffusion method was used to assess the antibiotic susceptibility of isolate EI6 to 11 different antibiotics. According to the data, it is susceptible to seven types and resistant to four (Table 3). The antibiotic resistance may be attributed to the cell wall structure, membrane permeability, and efflux mechanisms. EI6 showed susceptibility to vancomycin, and this is a fundamental property because vancomycin is the antibiotic of last resort in many cases, and it is one of the most effective antibiotics against multidrug-resistant pathogens causing clinical infections (Nami et al., 2014).

**Table 3:** Antibiotic susceptibility of *L. plantarum* EI6.

**Antibiotic**	**Dose (μg)**	**Susceptibility**
Ampicillin (AM)	10	R
Tetracycline (TE)	30	S
Cephradin (CE)	30	R
Naliixic (NA)	30	R
Amoxicillin (AX)	25	S
Ofloxacin (OFX)	5	S
Oxacillin (OX)	1	S
Erythromycin (E)	15	S
Ceftriaxone (CRO)	30	R
Tazobactam (TPZ)	110	S
Vancomycin (VA)	30	S

*S, susceptible; R, resistant*.

#### Low pH Resistance

**Figure 2:**
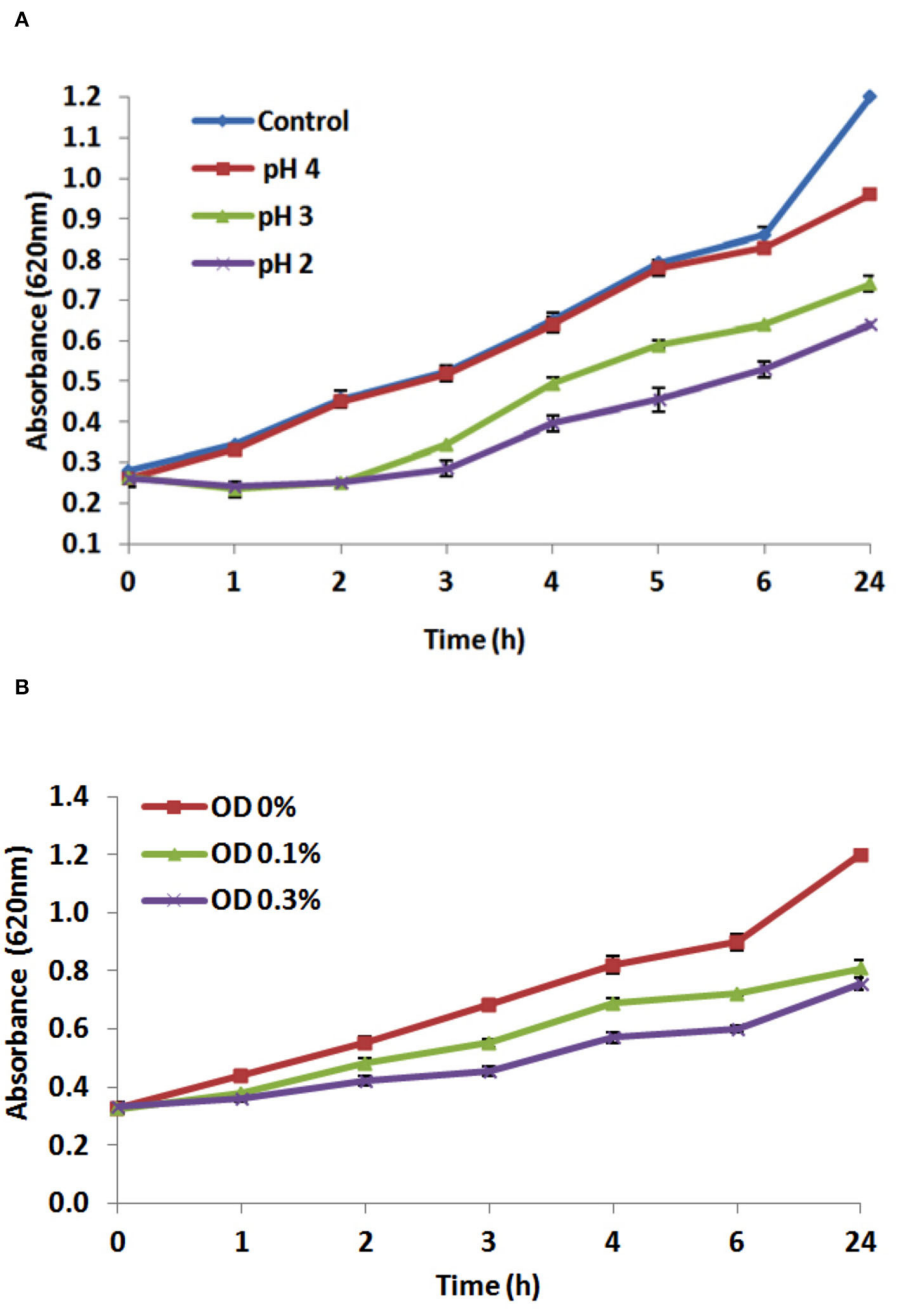
Effect of different pH values **(A)**, and effect of different bile salts concentrations (0.0, 0.1, and 0.3%) **(B)** on the growth of *L. plantarum* EI6 at 37°C for 24 h.

One of the major stress factors facing the ingested probiotics is the passage through the stomach and being subjected to gastric acids with a low pH value (Berrada et al., 1991). Therefore, one of the selection properties of new probiotic strains is the ability to withstand low pH (Cakir, 2003). The results demonstrated that *L. plantarum* EI6 is acid-tolerant as it was able to resist pH 4.0 and 3.0 for 4 h, which was indicated by increasing the O.D. values with survival rates of 100% and 77%, respectively, compared to the control (pH 6.2) (Figure 2A). Moreover, it managed to survive at pH 2.0 and increase the O.D. from 0.26 to 0.39 with a survival rate of 61.8% after 4 h. Further measurements at 6 and 24 h confirmed the acid-tolerance ability of isolate EI6 as it continued to grow and managed to increase the O.D. The ability of *L. plantarum* strains YO175 and OF101 to withstand low pH 2.5 and 2.0 for 4 h has been reported by Adesulu-Dahunsi (2018), as they did not show a substantial decrease in the viable cell count and their survival rates were over 97% (Adesulu-Dahunsi, 2018).

#### Bile Salts Resistance

The potential of *L. plantarum* EI6 to survive and proliferate in the presence of bile salts was investigated. For probiotics to survive in the intestine, they have to resist the antimicrobial conditions in the intestine, such as antimicrobial peptides, proteolytic enzymes, and bile salts that can destroy the bacterial cell membrane (Giles-Gómez et al., 2016). Although the bile salts concentration varies in the human digestive tract, the average concentration is thought to be 0.3%, and the staying time is believed to be 4 h (Gilliland et al., 1984). The influence of bile concentrations (0, 0.1, and 0.3% w/v) on the growth of the isolate EI6 is demonstrated in Figure 2B. *L. plantarum* EI6 was able to survive at 0.1% bile concentration, as detected by an increase in the O.D. from 0.3 to 0.68 (survival rate of 82.9%) after 4 h of incubation, and the growth was comparable to that of the control (MRS broth without bile salts). The isolate also showed good stability at 0.3% bile concentration, as the O.D. increased from 0.3 to 0.57 (survival rate of 69.5%) after 4 h of incubation. Subsequent measurements at 6 and 24 h validated isolate EI6's capacity to survive bile salts as it continued to grow and raise its O.D. The ability of LAB strains to survive high bile salt concentrations is due to the presence of a specific enzyme called bile salt hydrolase (BSH), which helps to hydrolyze conjugated bile salts and minimize their toxicity (Du Toit et al., 1998).

### Physicochemical Characterization of EI6-EPS

#### Total Carbohydrate Content (%)

The determination of the carbohydrate amount plays a crucial role not only to signify the purity of the produced exopolysaccharide but also to determine the EPS's functional properties and define its potential applications. In this study, the carbohydrate content was estimated as glucose, accounting for ~91.5% of the EI6-EPS. The sugar content was higher than that reported for the marine strain of *Rhodobacter johrii* CDR-SL 7Cii (86.82%) (Sran et al., 2019) but lower than the carbohydrate content of the exopolysaccharide from *Enterococcus faecalis* (94.7%) (Choudhuri et al., 2020).

#### Structural Functionalization by FTIR

**Figure 3:**
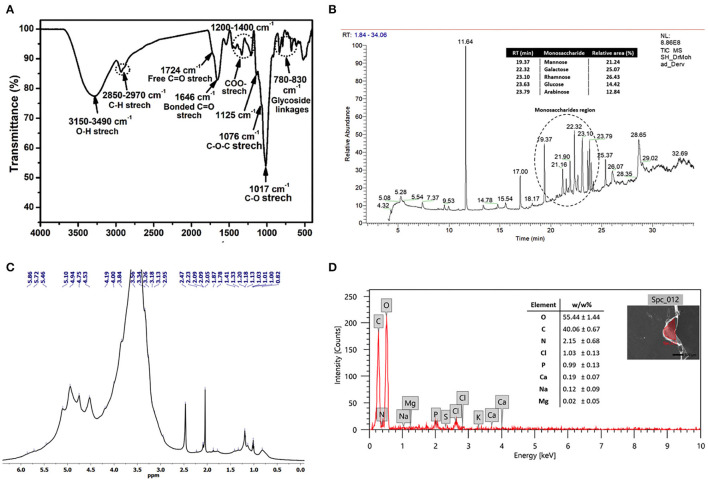
FTIR spectrum **(A)**, GC-MS chromatogram **(B)**, ^1^H NMR spectrum recorded at 323 K (2.0% w/v; DMSO-*d*_6_, 500 MHz) **(C)**, and SEM-EDX data of the produced exopolysaccharide (EI6-EPS) **(D)**.

Chemical functionalities of the polymeric EI6-EPS structure were identified through FTIR analysis. The IR spectrum revealed characteristic signatures and distinctive functional groups of carbohydrates (Figure 3A). Therefore, the assignments of the IR bands were mainly based on the previously reported polysaccharide spectra (Amer et al., 2020; Choudhuri et al., 2020). The broadband at 3,289 cm^−1^ in the region (3,150–3,490 cm^−1^) denoted the stretching vibration of a large number of hydroxyl (O-H) groups belonging to the sugar moieties of carbohydrates (Abid et al., 2018; Amer et al., 2020; Choudhuri et al., 2020; Sirin and Aslim, 2020). The absorption band at 2,970–2,850 cm^−1^ was assigned to the stretching vibration of the aliphatic C-H of the methyl or methylene groups in hexoses (e.g., galactose and glucose) and deoxyhexoses (e.g., rhamnose and fucose), which is characteristic of polysaccharides (Abid et al., 2018; Choudhuri et al., 2020; Sirin and Aslim, 2020). The absorption peaks at 1,724 and 1,656 cm^−1^ may relate to the stretching vibration of the C=O group in free and bonded states, respectively (Zhou et al., 2016; Abid et al., 2018; Sirin and Aslim, 2020). In contrast, the stretching vibration of C=O of the carboxyl (COO^−^) group was assigned to the region of 1,200–1,400 cm^−1^ (Sirin and Aslim, 2020).

The characteristic fingerprint region of the polysaccharide was ascertained from the bands in the area of 1,200–800 cm^−1^ (Shang et al., 2013; Abid et al., 2018; Sirin and Aslim, 2020). The intense band at 1,017 cm^−1^ and the shoulder at 1,076 cm^−1^ were distinctive and could be assigned to the stretching vibration of the C-O and C-O-C groups, respectively (Abid et al., 2018; Sirin and Aslim, 2020). Moreover, the small peak at 1,125 cm^−1^ indicated the existence of the sugar in a pyranose form (Abid et al., 2018). Finally, the peaks in the region of 830–780 cm^−1^ revealed the α- and β-glycoside linkages between sugar moieties (Coimbra et al., 1999).

#### Structural Study by ^1^H NMR

The NMR spectroscopic technique was used to get structural information on the EI6-EPS, such as monosaccharide composition, configurations, and linkage types (Li et al., 2011). The acquired ^1^H NMR spectrum of EI6-EPS (Figure 3C) revealed a similar signature to other reported polysaccharides (Amer et al., 2020; Choudhuri et al., 2020). The ring protons at the positions C2–C6 belong to the sugar moieties and appeared in the range of δ = 2.96–4.19 ppm, while the anomeric protons displayed in the range of δ = 4.45–5.16 ppm. The broadening of the H_2_O signal at 3.3 ppm hindered the resolution and assignment of the monosaccharide ring protons. The existence of β- and α-glycosidic linkages between sugar moieties was investigated from the resonance peaks at δ = 4.45–4.75 and δ = 4.84–5.16, respectively (Agrawal, 1992), which agreed with the FTIR data. The configuration and mode of the glycosidic bonds between sugars could not be identified due to the broadness of the anomeric protons. As determined by GC-MS analysis, the shoulder peaks within the range of 3.43–4.24 ppm suggested the presence of glucose and galactose in the EPS structure (Sivasankar et al., 2018).

The existence of β- and α-glycosidic linkages between sugar moieties was investigated from the resonance peaks at δ = 4.45–4.75 and δ = 4.84–5.16, respectively (Agrawal, 1992), which agreed with the FTIR data. The configuration and mode of the glycosidic bonds between sugars could not be identified due to the broadness of the anomeric protons. The shoulder peaks within the range of δ = 3.43–4.24 ppm proposed the existence of glucose and galactose in the EPS structure (Sivasankar et al., 2018) as determined by GC-MS analysis. It has been reported that the alkyl group region lies in the range of δ =1.2–2.3 ppm (Wang et al., 2014). The presence of signals at δ = 1.13–1.21 ppm denoted the methyl (–CH_3_) protons of the fucose and rhamnose moieties (Agrawal, 1992; Choudhuri et al., 2020).

#### Monosaccharide Composition by GC-MS

The monosaccharide units that construct the structure of EI6-EPS were identified using GC-MS analysis with the help of the NIST library (Olasehinde et al., 2019). The analysis indicated that EI6-EPS is composed of different sugar moieties, suggesting that it is a heterogeneous polysaccharide (Sirin and Aslim, 2020). Most of the peaks in the GC chromatogram were identified as monosaccharide derivatives by the MS detector with the help of the NIST library. The GC-MS data analysis demonstrated that the EI6-EPS comprises at least five monosaccharides investigated consecutively (time = 19–24 min), denoting the most intense region in the GC chromatogram. The comparable area% was calculated based on the relative abundances of these moieties, giving the order rhamnose > galactose > mannose > glucose > arabinose with a relative area% of 26.43, 25.07, 21.24, 14.42, and 12.84, respectively (Figure 3B). The sugar moieties exist mainly in the pyranose with less existence of the furanose form. EI6-EPS was very similar to the sugar composition of the EPS produced by *Rhodobacter johrii* CDR-SL 7Cii (Sran et al., 2019), *Enterococcus faecium* WEFA23-2 (Jia et al., 2019), *L. delbrueckii* ssp. *bulgaricus* B3, and *L. plantarum* GD2 strains (Sirin and Aslim, 2020), but with different molar ratios.

#### Morphological and Elemental Studies by SEM and EDX Spectroscopy

**Figure 4:**
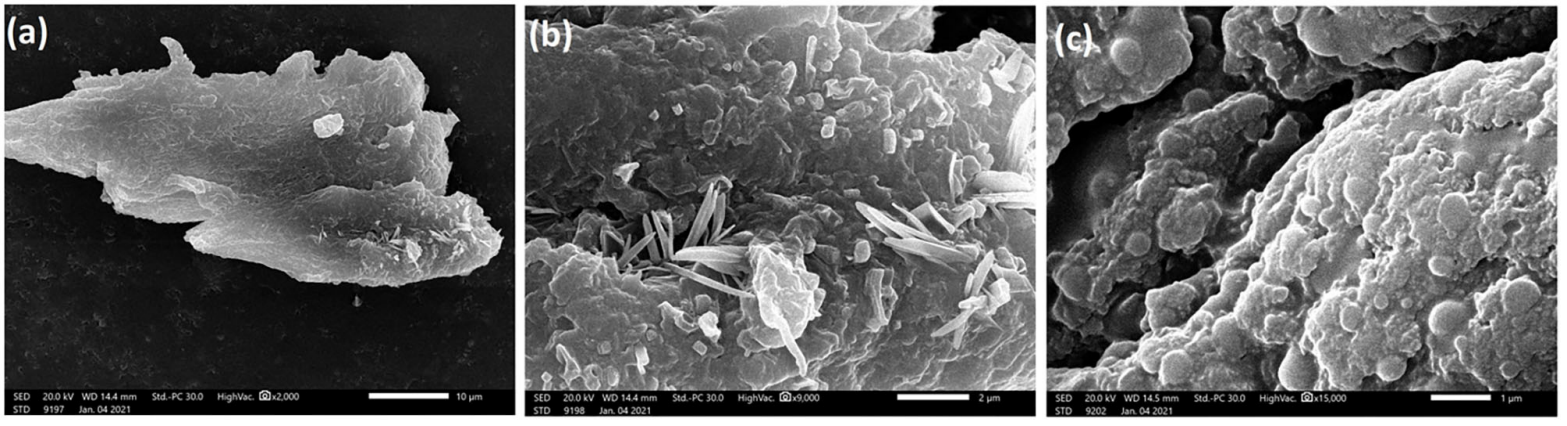
SEM images of the produced exopolysaccharide (EI6-EPS) at 2,000× **(A)**, 9,000× **(B)**, and 15,000× **(C)**.

A scanning electron microscope is a valuable tool to visualize the surface topography of materials such as polymers (Ahmed et al., 2013). The SEM micrographs of the EI6-EPS revealed a rough and irregular surface (Figure 4) that resembled the reported morphology for the exopolysaccharides from *L. delbrueckii* ssp. *bulgaricus* B3 and *L. plantarum* GD2 strains (Sirin and Aslim, 2020).

The elemental analysis utilizing EDX analysis revealed the predominance of oxygen and carbon with a mass ratio of 55.44 and 40.06 (w/w%), respectively. The high mass% of those two elements emphasized that the purified EI6-EPS is comprised mainly of carbohydrates (Sirin and Aslim, 2020) (Figure 3D). This result is consistent with the high carbohydrate content (91.5%) as determined by UV-vis analysis. The presence of nitrogen (2.15 w/w%) and phosphorus (0.99 w/w%) proposed the lack of protein and phospholipids accompanied by the purified EI6-EPS. Other elements including chloride (1.03%), sodium (0.12%), calcium (0.19%), and magnesium (0.02%) were also detected; however, the sulfur was null. The elemental composition of the EI6-EPS was similar to the EPS produced by *Rhodobacter johrii* CDR-SL 7Cii (Sran et al., 2019), *L. delbrueckii* subsp. *bulgaricus* NCFB 2483 (Goh et al., 2005), *L. delbrueckii* ssp. *bulgaricus* B3, and *L. plantarum* GD2 (Sirin and Aslim, 2020).

#### Cytotoxicity Assay

The cytotoxicity of EI6-EPS was evaluated against human dermal fibroblast cell lines. The EPS had no cytotoxic effects against HDF cell lines at low concentrations and the IC50 was determined to be > 100 μg/ml as the cells showed only a very minimal decrease in the viability at concentrations as high as 100 μg/ml, which indicates the safety of EI6-EPS toward the skin cells.

#### *In vitro* Wound Healing Activity

**Figure 5:**
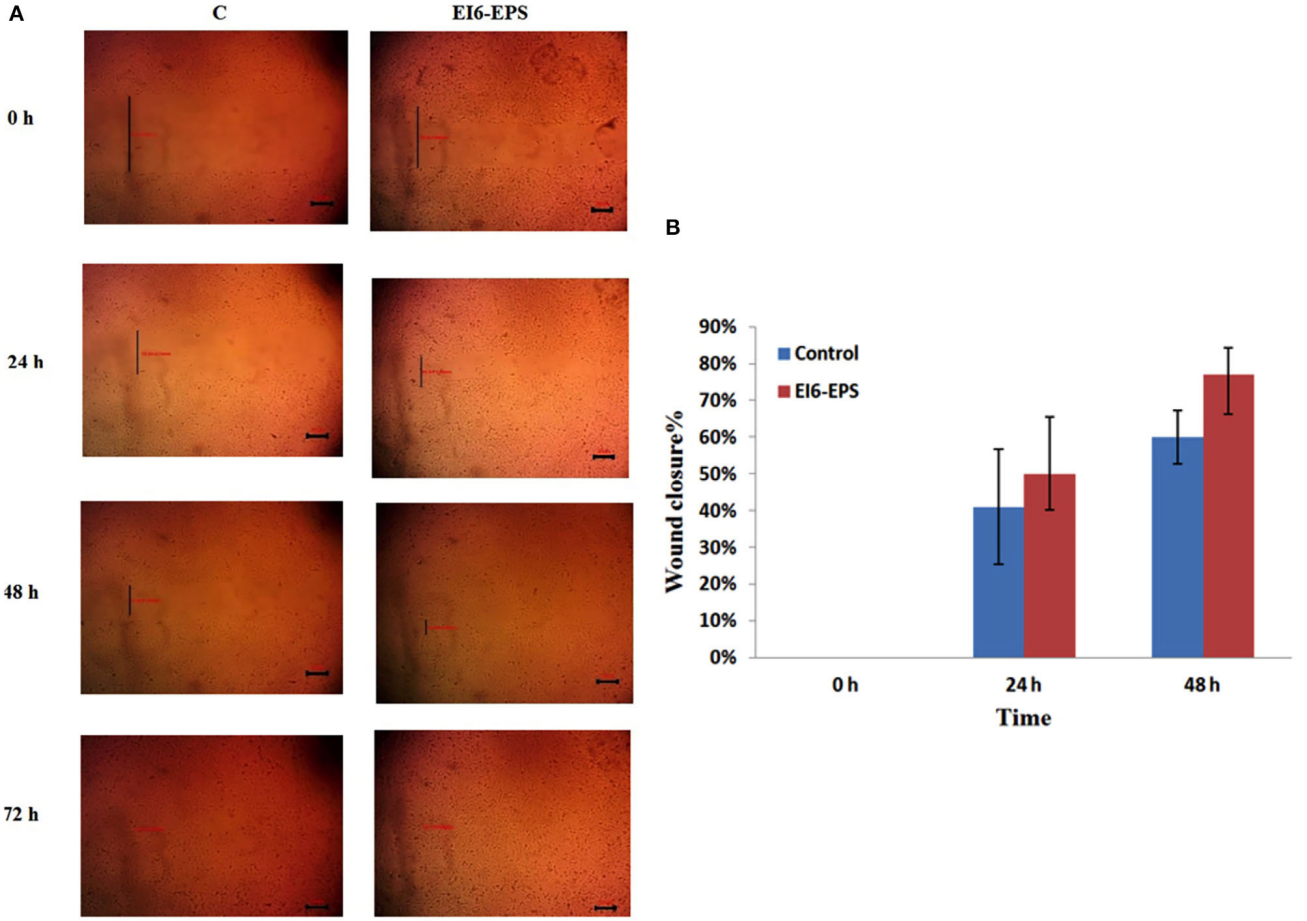
*In vitro* cell migration of skin fibroblasts by EI6-EPS scratch was created in monolayer of HSF cells and was treated with EI6-EPS. Control group was without any treatment. **(A)** Photographs of wound area treated with EI6-EPS at different time intervals were taken using an inverted microscope. **(B)** Percentage wound closure in control and treated cells at different time intervals.

The proliferative effect of EI6-EPS on the HSF cell line was assessed using the wound scratch assay (Figure 5). Wound width was calculated as the average distance between the edges of the scratches. The wound width decreases as cell migration is induced.

The *in vitro* HSF cell line scratch assay revealed that EI6-EPS significantly induced cell migration. After 24 h, EI6-EPS–treated cells showed 50% wound closure compared to 41% in the control group, while at 48 h, EI6-EPS–treated cells showed 77% wound closure compared to 60% in the control group.

These findings indicate that the EI6-EPS has wound healing and cell migration bioactivity, making it suitable for a variety of therapeutic and pharmacological applications.

The role of EI6-EPS in the healing process may be attributed to the thought that various cell surface receptors can identify and bind to β-glucan because of its triple-helical conformation, causing inflammatory cytokines or other modulators to be activated, as well as its antioxidant activity (Weber et al., 2016; Trabelsi et al., 2017; Sahana and Rekha, 2019). However, the exact mechanism of action needs to be investigated further utilizing *in vivo* models.

The idealistic compound for wound healing should fulfill a variety of current global needs while also allowing for uneventful and scar-free accelerated healing. The EI6-EPS is a safe, natural, and biocompatible polymer with a cell migration improvement activity, and its structural properties are suitable for therapeutic and pharmacological applications, making it an excellent bioactive compound for use in wound care. The wound healing activity of *Lactiplantibacillus* sp. has been documented by Trabelsi et al. (2017).

The chemical analysis confirmed the high purity of the EI6-EPS produced by *L. plantarum* EI6, as noticed by the high carbohydrate content (>90%) using the sulfuric acid method. FTIR and 1D NMR emphasized the polysaccharide nature of EI6-EPS through the characteristic curve and peaks of the recorded spectra. GC-MS results proposed that EI6-EPS is a heterogenous polysaccharide composed mainly of five monosaccharides. The outcomes of this study suggest that the polysaccharides, specifically those rich in galactose, mannose, and glucose, significantly improve the wound-healing properties of EI6-EPS (Mapoung et al., 2021) and that EI6-EPS enhances keratinocyte and fibroblast cell proliferation (Krupodorova et al., 2015; Veeraperumal et al., 2020). These findings are also in agreement with previous studies concerned with the wound-healing potential of polysaccharides derived from different sources such as seaweed (*Gracilaria lemaneiformis*) (Veeraperumal et al., 2020), plant (*Bletilla striata* and *Astragalus membranaceus*) (Zhao et al., 2017; Zhang et al., 2019), or mushrooms (*Ganoderma lucidum, Agaricus blazei* and *Phellinus gilvus*) (Hu et al., 2019).

## Conclusion

In this study, a new marine LAB isolate, *L. plantarum* EI6, was evaluated for its probiotic potential besides production and primary characterization of its EPS. The obtained data revealed that this isolate exhibits promising probiotic and technological properties by their ability to withstand low pH, high bile salt concentrations, and broad antibacterial activity. Furthermore, the purified EI6-EPS is a heteropolysaccharide, with a high carbohydrate content (~91.5%) that associates with the predominance of oxygen (55.44 w/w%) and carbon (40.06 w/w%) in EDX analysis, indicating efficient purification of the polysaccharides. FTIR and NMR emphasized the polysaccharide nature of the EI6-EPS, as illustrated by the recorded characteristic peaks. GC-MS identified five abundant monosaccharides in the pyranose form (i.e., rhamnose, galactose, mannose, glucose, and arabinose) connected by α- and β-glycosidic linkages. EI6-EPS has been proven to be safe on HDF cell lines and stimulates the proliferation and migration of HSF. These findings suggest that *L. plantarum* EI6 may be employed as a safe source for bioactive compounds for pharmacological applications. However, more *in vivo* research is needed to prove the beneficial effects in the wound care sector.

## Data Availability Statement

The original contributions presented in the study are included in the article/supplementary material, further inquiries can be directed to the corresponding author/s.

## Author Contributions

EZ and MI are equally contributed in concept, design, analysis of data, and writing the manuscript. All authors contributed to the article and approved the submitted version.

## Conflict of Interest

The authors declare that the research was conducted in the absence of any commercial or financial relationships that could be construed as a potential conflict of interest.

## Publisher's Note

All claims expressed in this article are solely those of the authors and do not necessarily represent those of their affiliated organizations, or those of the publisher, the editors and the reviewers. Any product that may be evaluated in this article, or claim that may be made by its manufacturer, is not guaranteed or endorsed by the publisher.
